# Supplementary material for: Rapid accelerations of Antarctic Peninsula outlet glaciers driven by surface melt
Source: Nat Commun. 2019 Sep 20;10:4311. doi: 10.1038/s41467-019-12039-2 (PMC6754444; doi:10.1038/s41467-019-12039-2)
Supplement: Supplementary file 1 — Supplementary Information [file 41467_2019_12039_MOESM1_ESM.pdf]

Supplementary Information for "Rapid accelerations of Antarctic Peninsula outlet glaciers driven by surface melt"

Tuckett et al.

Supplementary Figures:

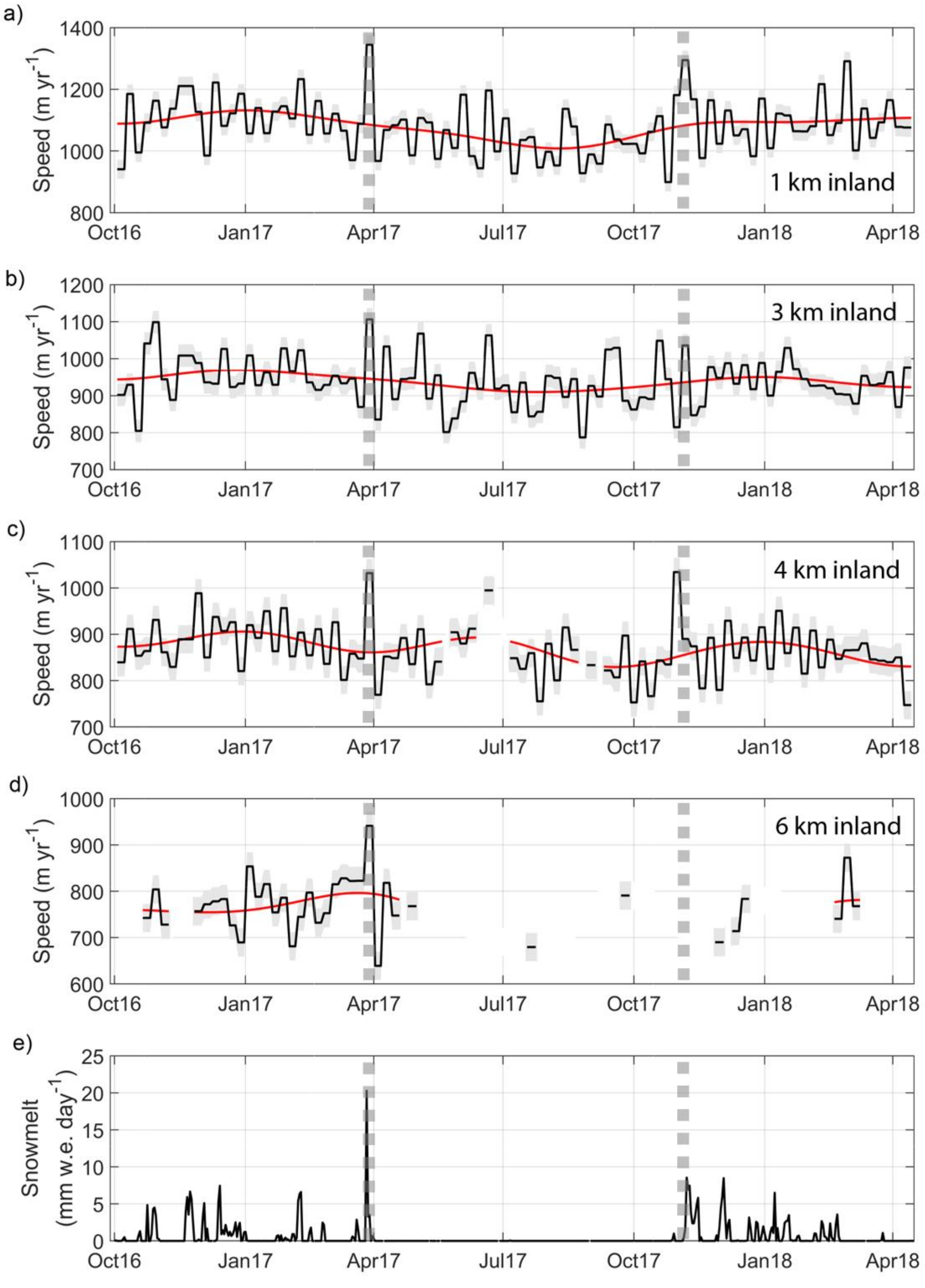

Supplementary Figure 1. Velocity of different ROIs on Crane Glacier and modelled snowmelt. Note the concurrence of melt and speed-up events. The red line is a running mean of 144 days and velocity uncertainties are shown by shaded envelopes around the data. Dashed lines indicate events interpreted as meltwater induced speed-ups.

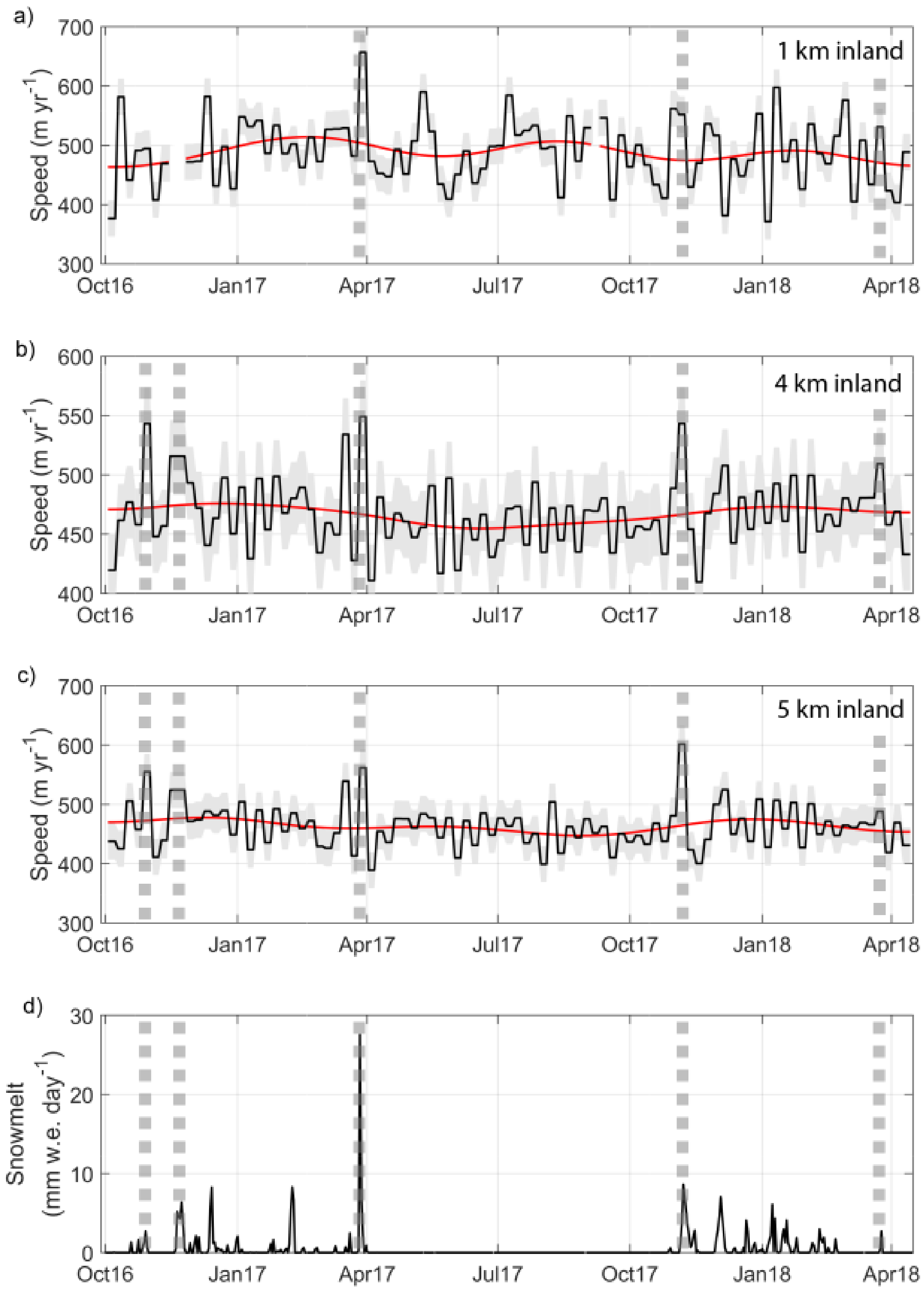

Supplementary Figure 2. Velocity of different ROIs on Jorum Glacier and modelled snowmelt. Note the concurrence of melt and speed-up events. Red line is a running mean of 144 days and velocity uncertainties are show by shaded envelopes around the data. Dashed lines indicate events interpreted as meltwater induced speed-ups.

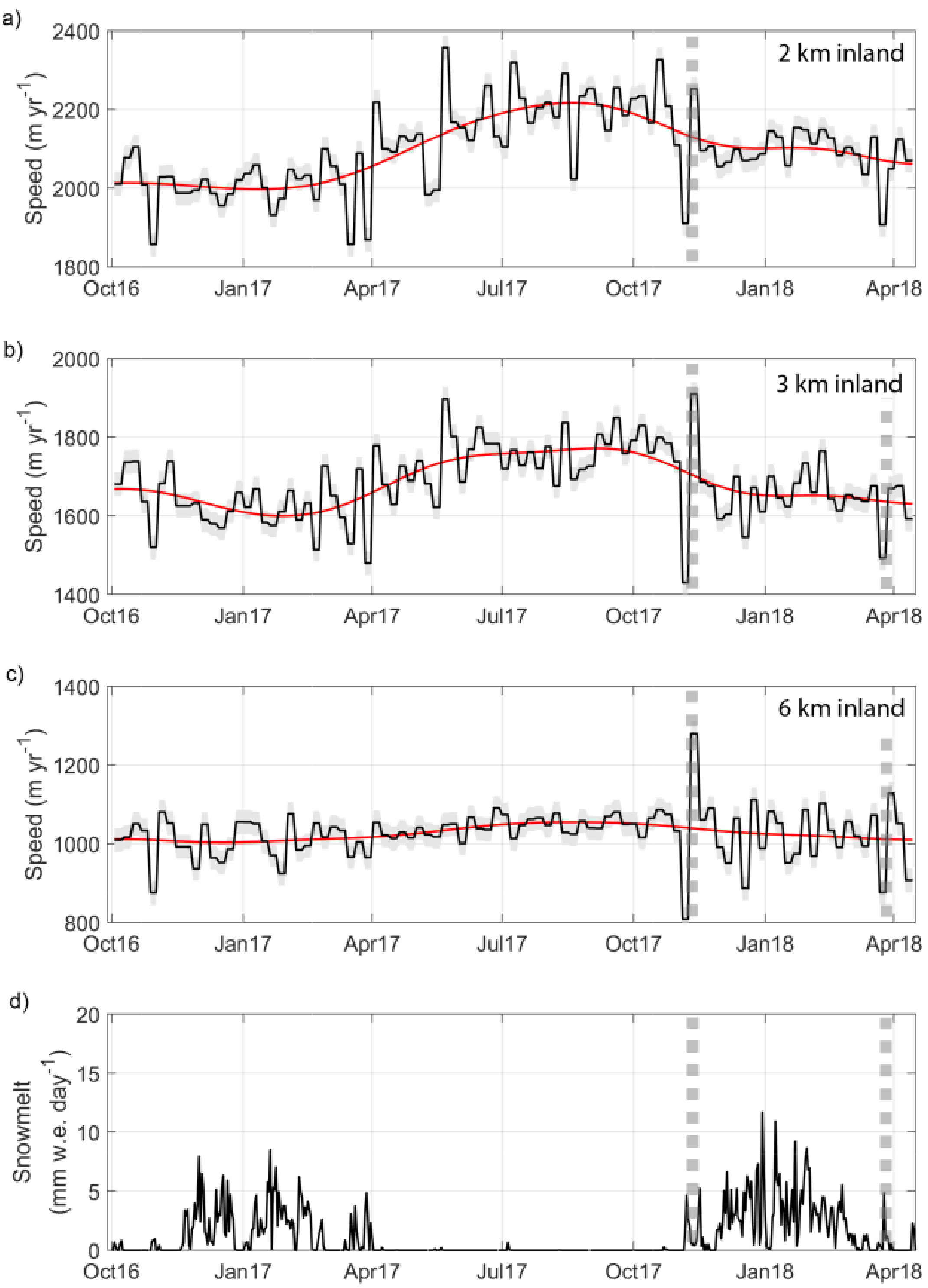

Supplementary Figure 3. Velocity of different ROIs on Cayley Glacier and modelled snowmelt. Note the increase in relative magnitude of speed-up events inland and the concurrence of melt and speed-up events. Red line is a running mean of 144 days and velocity uncertainties are show by shaded envelopes around the data. Dashed lines indicate events interpreted as meltwater induced speed-ups.

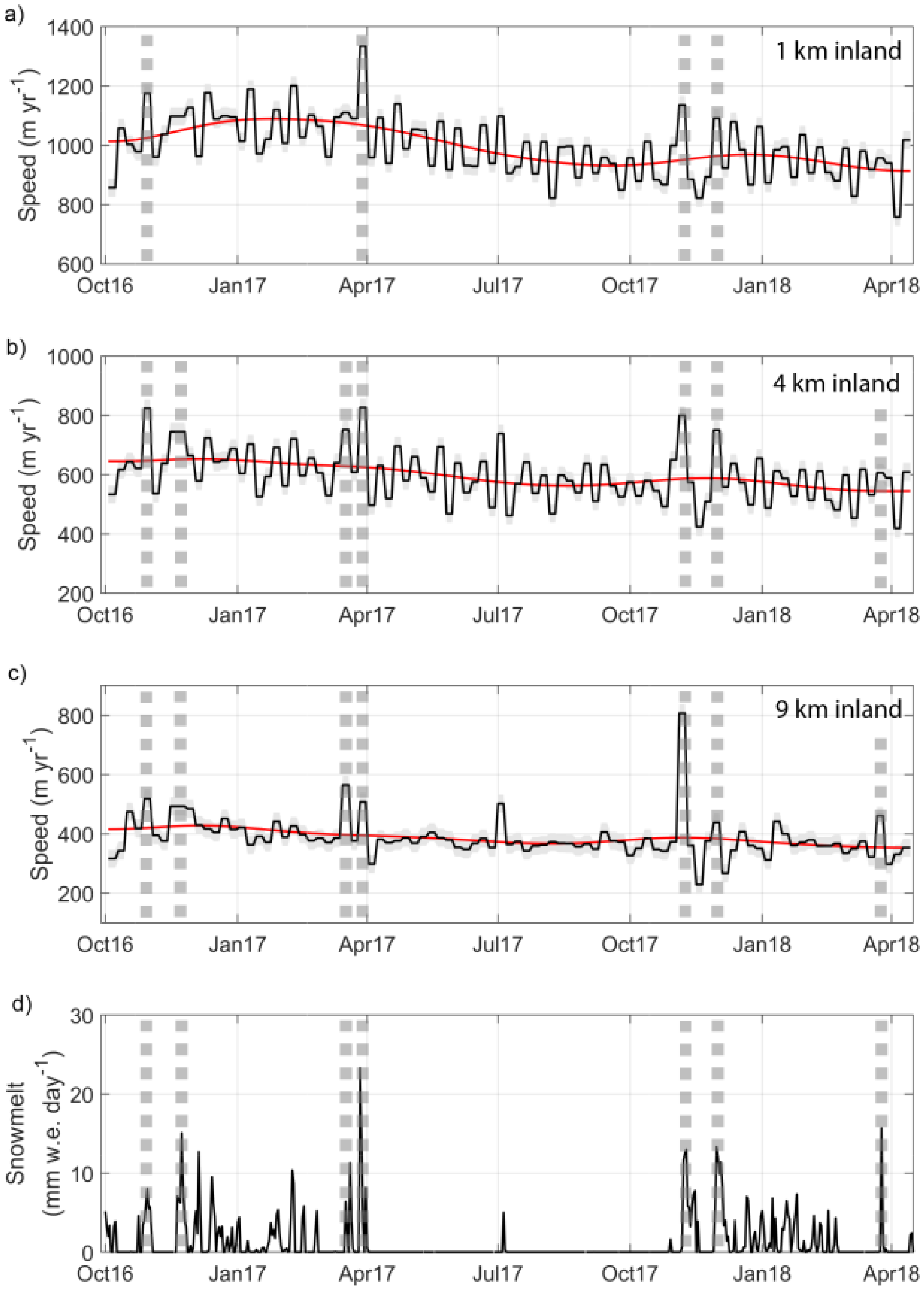

Supplementary Figure 4. Velocity of different ROIs on Hektoria Glacier and modelled snowmelt. Note the concurrence of melt and speed-up events. Red line is a running mean of 144 days and velocity uncertainties are show by shaded envelopes around the data. Dashed lines indicate events interpreted as meltwater induced speed-ups.

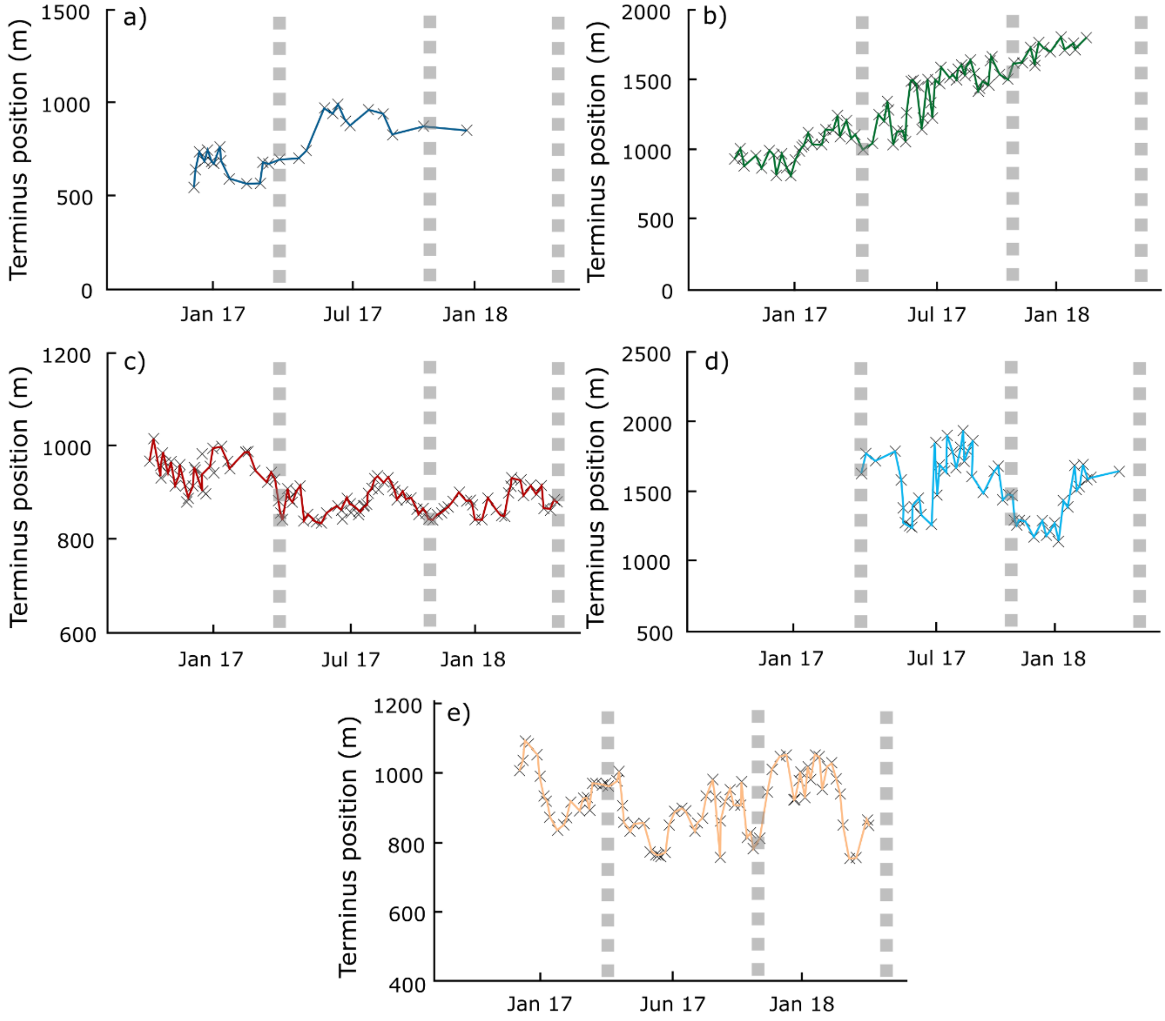

Supplementary Figure 5. Variations in calving front position of the studied glaciers. a) Crane; b) Jorum; c) Cayley; d) Hektoria; e) Drygalski. Vertical dashed lines indicate the timing of glacier-wide speed-up events identified in Fig. 2. Positions are width averaged, defined as the mean distance from the glacier centreline (i.e. increases in terminus position indicate an advance).

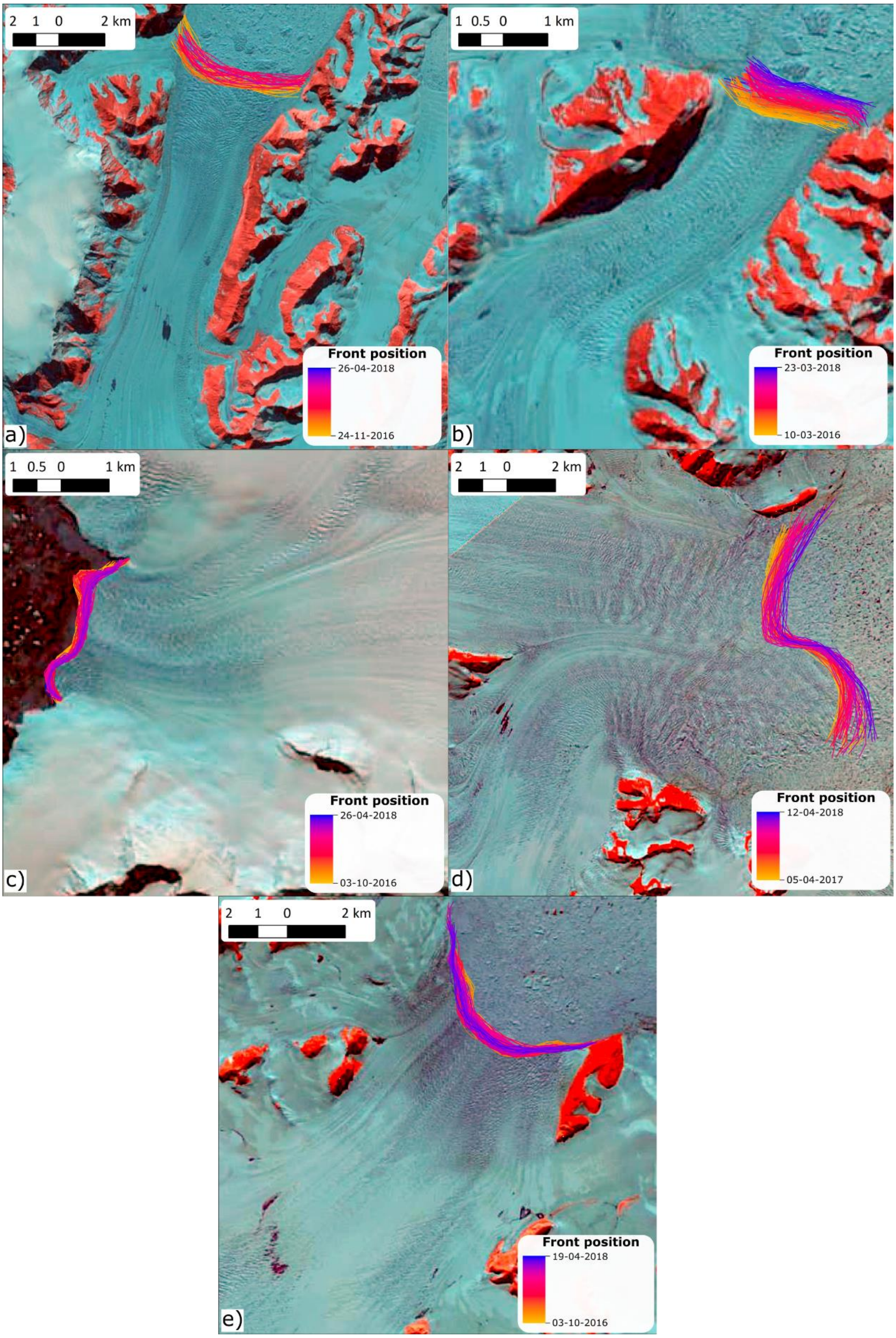

Supplementary Figure 6. Mapped front positions of studied glaciers. a) Crane; b) Jorum; c) Cayley; d) Hektoria; e) Drygalski. Background is a Landsat 8 image. Image accessed from USGS Earth Explorer (https://doi.org/10.5066/F71835S6).

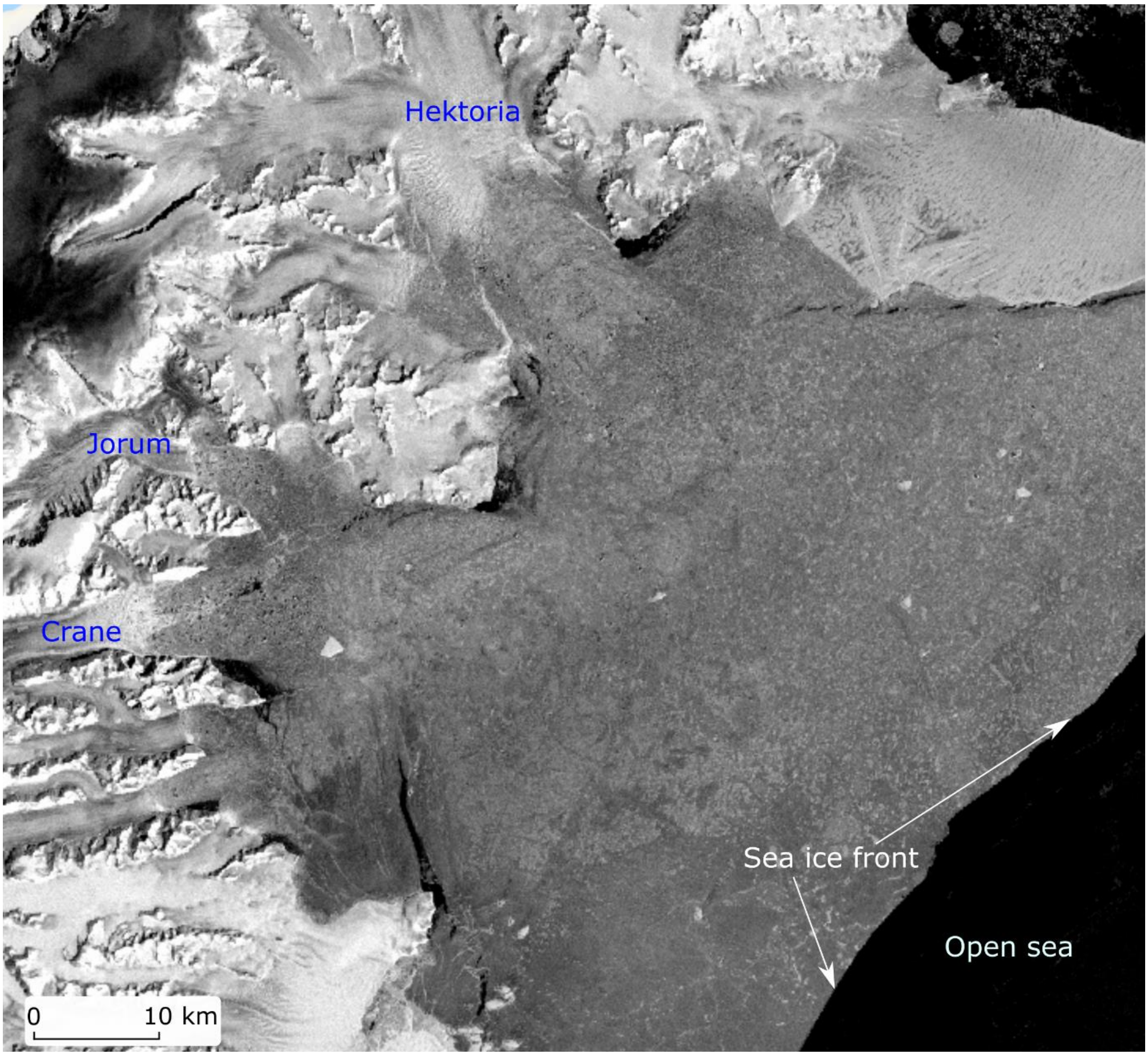

Supplementary Figure 7. Location of sea ice front on 07-04-2017, the end of the melt season, in relation to 3 of the studied glaciers. Image is from Sentinel 1, accessed in Google Earth Engine ©.

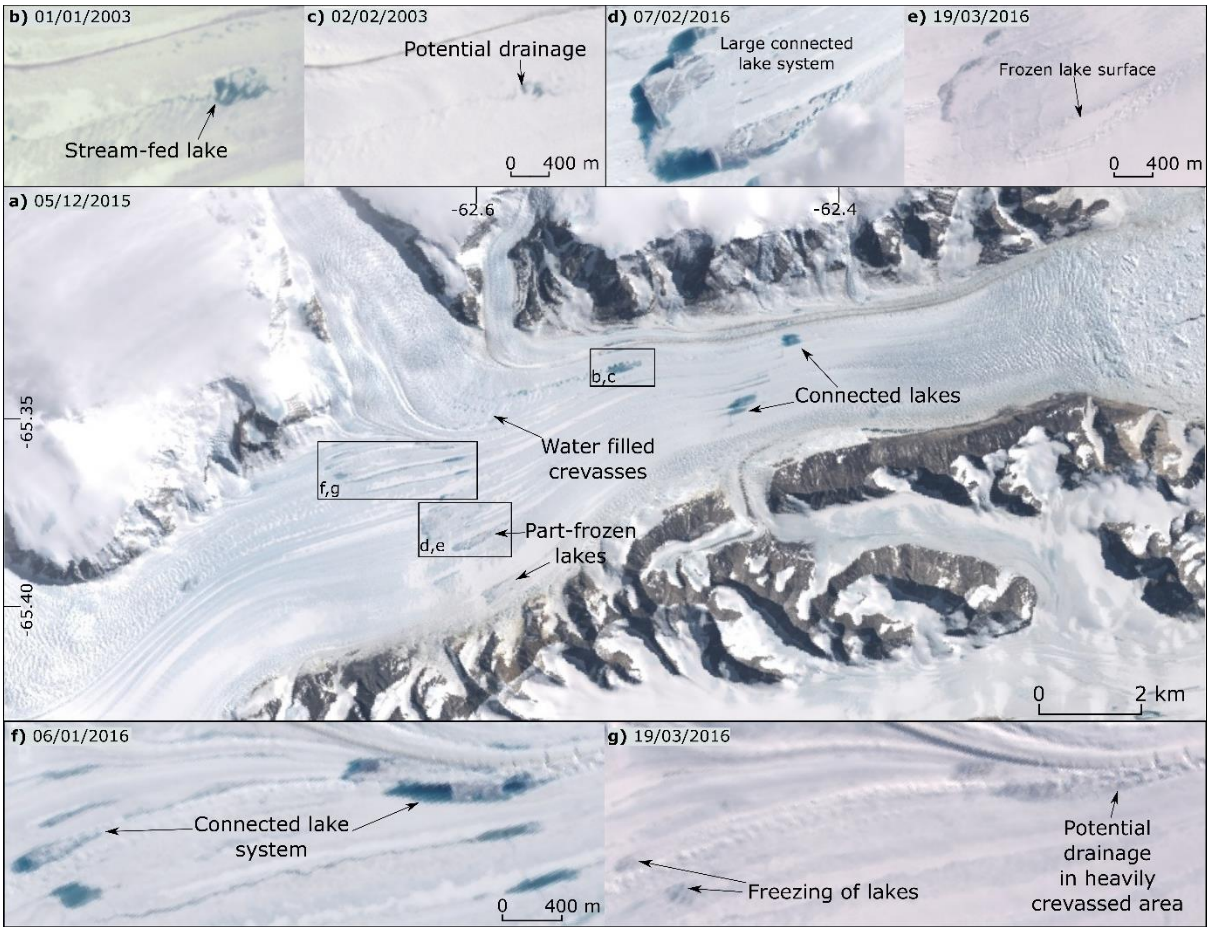

Supplementary Figure 8. Surface meltwater features of Crane Glacier. a, Landsat 8 image of meltwater features on Crane Glacier. b,c, Landsat 7 images of a surface lake fed by a meltwater stream which potentially drained. Note the lack of a frozen smooth surface surrounding the lake. d,e. Landsat 8 images of a connected lake system upglacier. These lakes were observed to freeze at the end of the melt season. f,g, Landsat 8 images of a connected lake system. Many of the lakes freeze, as indicated by frozen silhouettes (g). However, a lake in a heavily crevassed area at the bottom of the meltwater system may have drained, as indicated by the lack of any frozen imprint. All images accessed in Google Earth Engine ©.

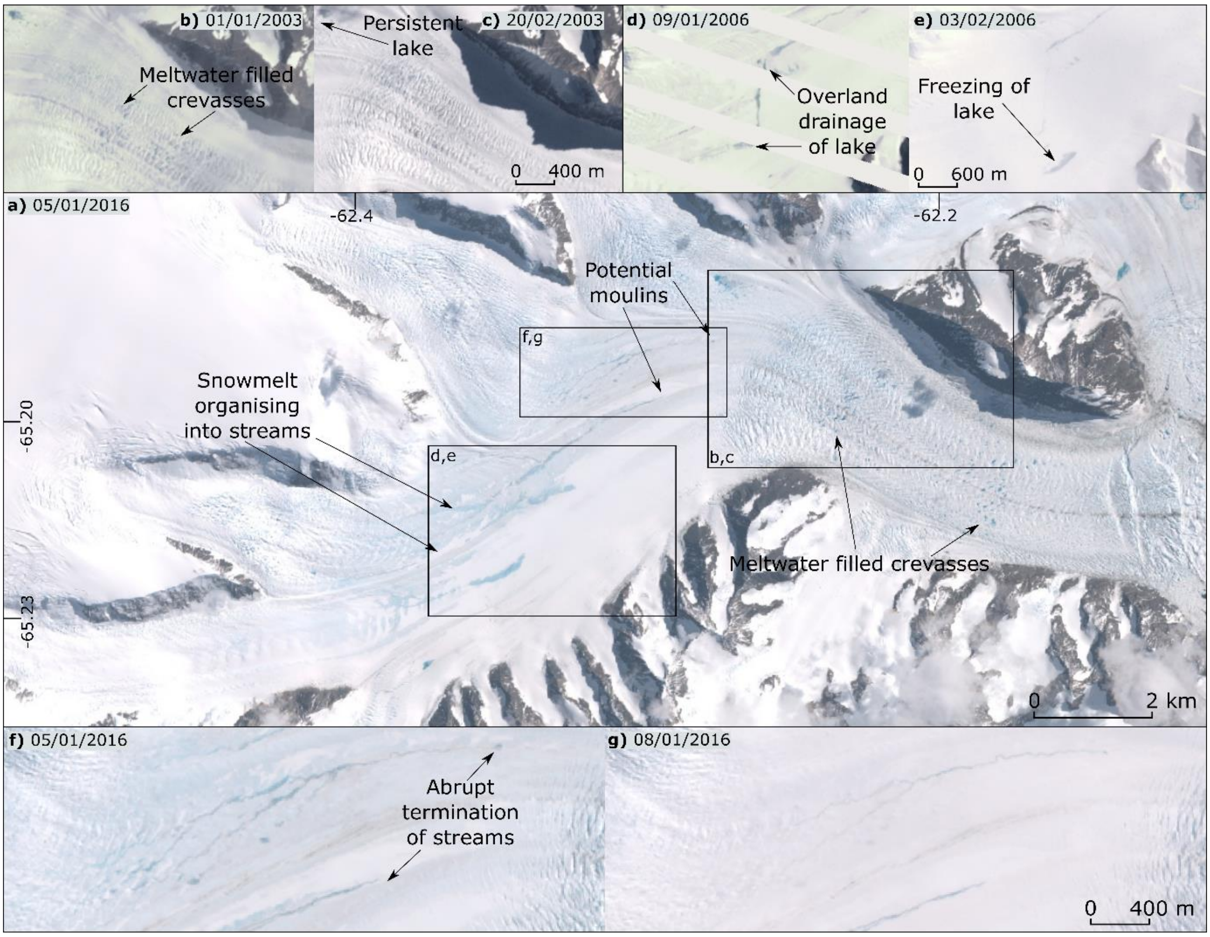

Supplementary Figure 9. Surface meltwater features of Jorum Glacier. a, Sentinel 2 image of Jorum Glacier showing an overview of surface meltwater features. b,c, Landsat 7 images of meltwater filled crevasses which appear to drain between b and c. Note that a lake further upglacier, at higher elevations, is maintained at this time, suggesting that the water in the crevasses is not freezing. d,e, Landsat 7 images of connected lakes which later freeze. Note the regions of no data are a consequence of the scan-line corrector failure. f,g, Sentinel 2 images of meltwater streams which end abruptly. The terminal position of these streams is persistent throughout the melt season, and recurrent between different years. We interpret these terminations as likely locations of moulins. All images accessed in Google Earth Engine ©.

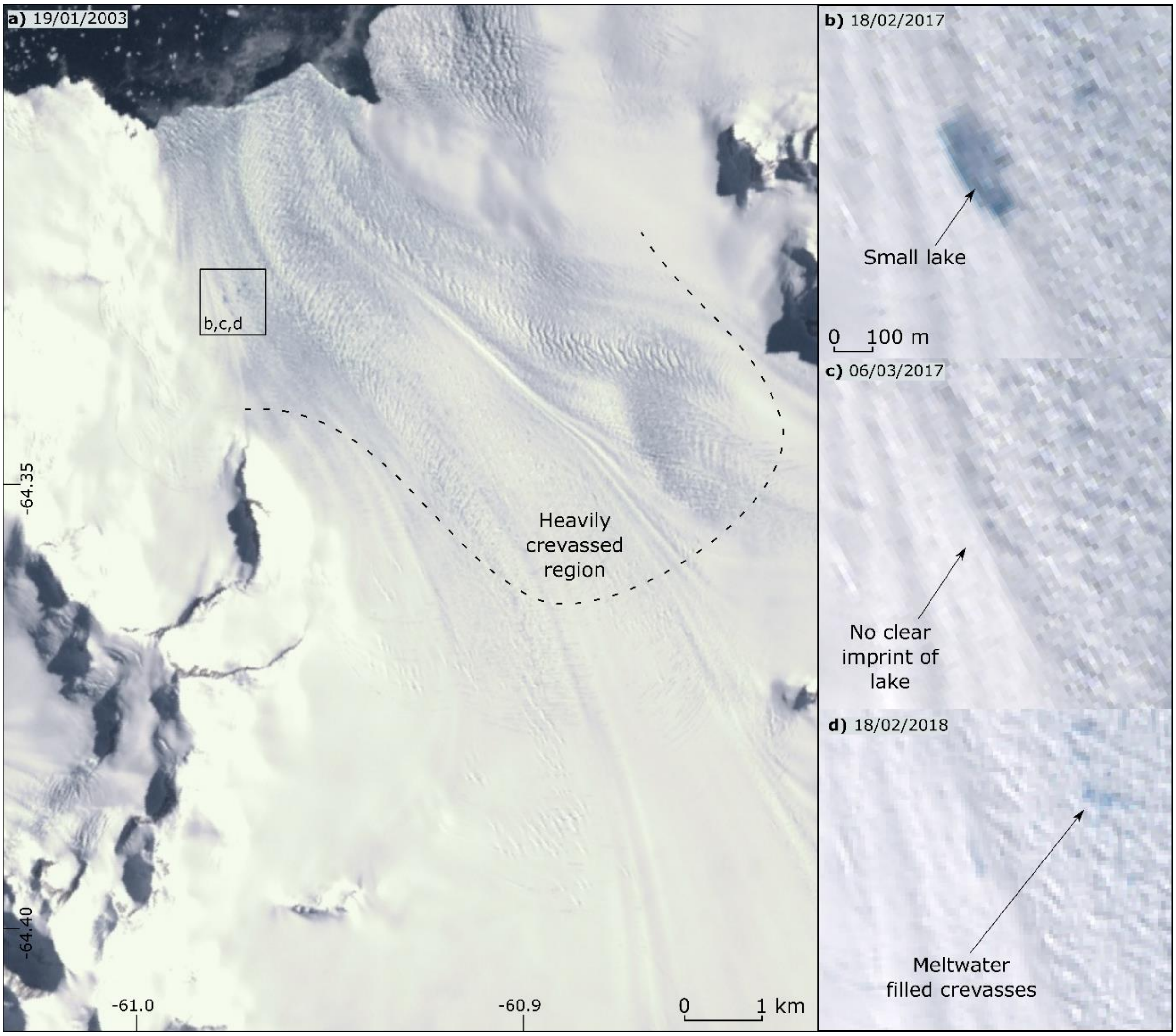

Supplementary Figure 10. Surface meltwater features of Cayley Glacier. a, Landsat 7 image providing an overview of Cayley Glacier. Note that the region beyond the dashed line is heavily crevassed. This terminal, lower altitude, region is the likely area where melt is produced. Any water produced here is therefore likely to go into crevasses. b,c Landsat 8 images of a small lake which regularly occurs on Cayley Glacier. This lake may drain, due to the lack of a clear frozen imprint. d, A later Sentinel 2 image shows meltwater in neighbouring crevasses and the beginning of lake formation. All images accessed in Google Earth Engine ©.

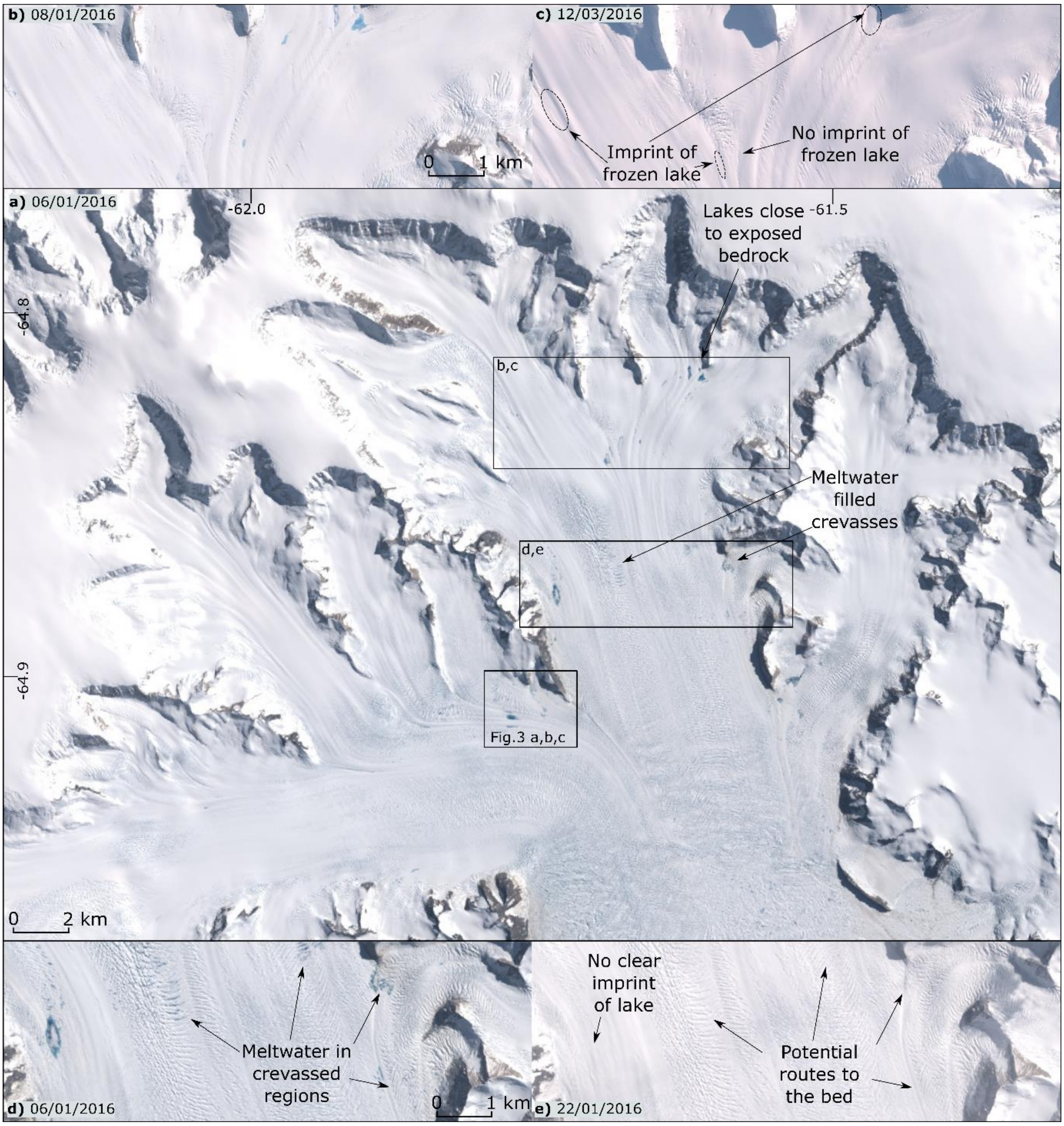

Supplementary Figure 11. Surface meltwater features of Hektoria Glacier. a) Landsat 8 image showing an overview of surface meltwater features on Hektoria Glacier. Note that the location of Figure 3 a,b and c is shown. b,c, Landsat 8 image showing lakes high up on the glacier. Many of these lakes freeze, as evidenced by their smooth imprint. However, one lake leaves no such imprint and may have drained. d,e, Sentinel 2 images showing a region where water potentially drains to the bed. Meltwater is evident in crevasses in d, but has potentially been routed to the bed in e. The lake in d leaves no imprint of a frozen surface visible in e. All images accessed in Google Earth Engine ©.

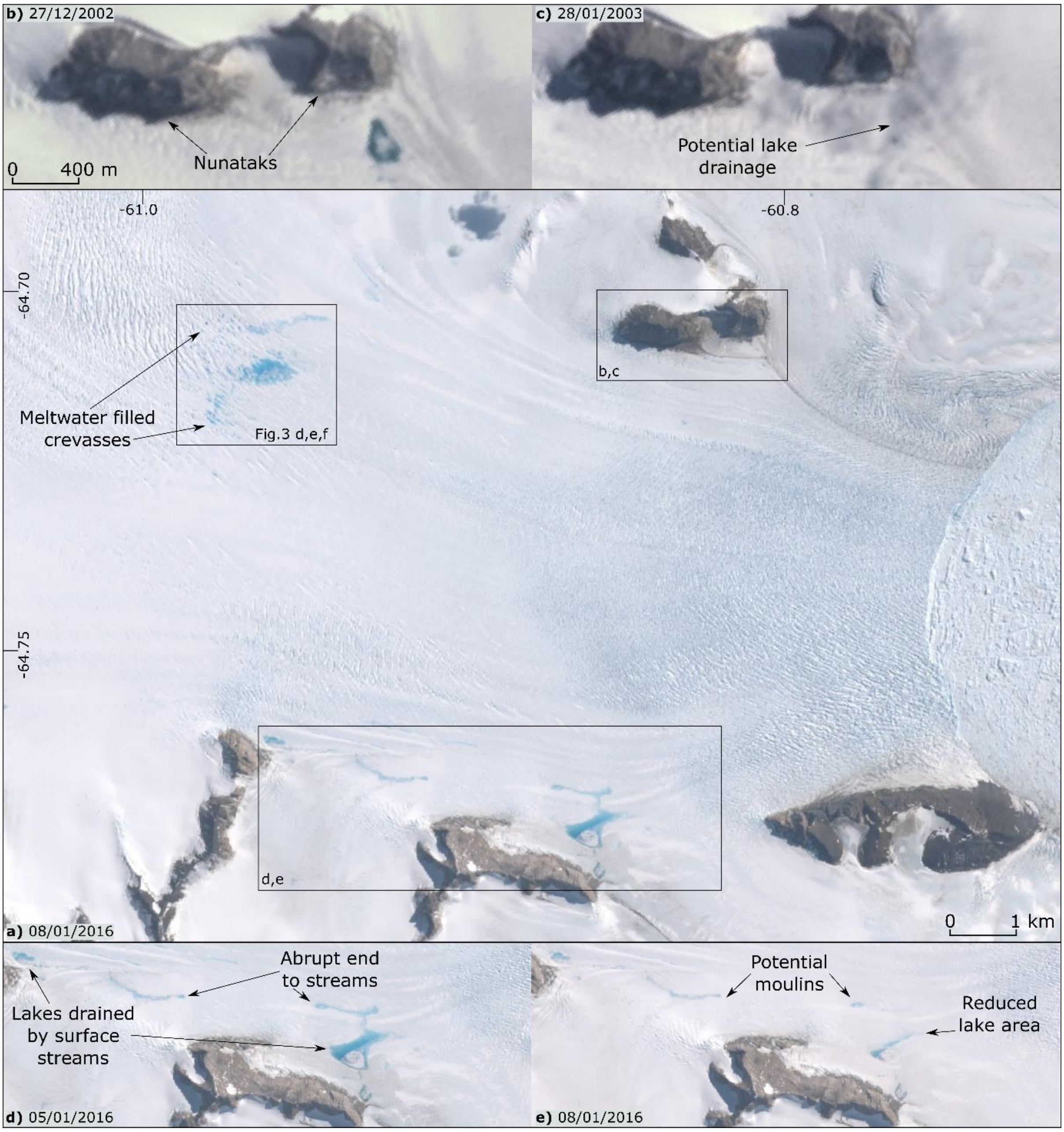

Supplementary Figure 12. Surface meltwater features of Drygalski Glacier. a, Sentinel 2 image of Drygalski Glacier, providing an overview of common meltwater features. Note the location of Figure 3 d,e and f is shown. b,c, Landsat 7 imagery of a lake which potentially drains to the glacier bed, located near a nunatak. The lake is partially obscured by cloud in c, but no clear imprint of a frozen lake is evident. d,e, Sentinel 2 imagery of surface meltwater systems on the southern side of Drygalski. The lakes are drained by surface streams which abruptly end. Lake area is reduced in e, but the stream is maintained and there is no sign of freezing. We suggest that the termination of these streams are potential moulins. All images accessed in Google Earth Engine ©.
